# Weight Loss Medication Prescription Prevalence in the Active Component, 2018-2023

**Published:** 2024-01-20

**Authors:** Nathan C. Lorei, Shauna L. Stahlman, Gi-Taik Oh, Natalie Y. Wells

**Affiliations:** 1 Uniformed Services University of the Health Sciences, Bethesda, MD; 1 Armed Forces Health Surveillance Branch, Public Health Division, Defense Health Agency, Silver Spring, MD

## Abstract

The U.S. military has witnessed rising obesity among active component service members. The Department of Defense authorized coverage of weight loss medications in 2018, but no study has evaluated prescription prevalence within the active component. This descriptive retrospective cohort study analyzed data from active component U.S. military service members from January 2018 through June 2023. The study used data from the Defense Medical Surveillance System to determine prescription period prevalence of weight loss medication. Data on demographics, body mass index, and history of diabetes were considered. The study revealed a 100-fold increase in the prescription period prevalence of weight loss agents in the active component from their initial authorization date. Demographics associated with higher prescription period prevalence were non-Hispanic black race and ethnicity, female sex, and older age. Service members in the health care occupations and the Navy had higher rates compared to other service branches and occupations. The findings indicate a significant rise in the period prevalence of weight loss prescriptions over time. Further research is recommended to assess the effectiveness, safety, and use in austere military environments.

**What are the new findings?:**

Since approval for active component use in 2018, the prevalence of weight loss prescriptions in the active-component increased nearly 100-fold, from 1.2 to 104.1 per 100,000 service members. Demographic categories associated with increased prescription rates include female sex, older age, non-Hispanic Black race and ethnicity, Naval service, and working in health care-associated occupations.

**What is the impact on readiness and force health protection?:**

The use of weight loss medications will likely continue to increase given the ongoing obesity epidemic in the U.S. Further study evaluating their real world effectiveness in weight management and safety for use among service members in austere and deployed environments should be considered.

## 1 BACKGROUND

In 2000, the World Health Organization designated obesity a global epidemic[1]. The U.S. faces an increasing prevalence of obesity, which affects both the general population and the armed forces. [2] The prevalence of obesity among active component service members (ACSM) rose from 16.1% in 2018 to 18.8% in 2021.3 Furthermore, the combined prevalence of both obesity and overweight increased from 65.5% in 2018 to 67.3% in 2021. [3] Obesity within military ranks not only compromises readiness and functional capabilities but also correlates with various musculoskeletal injuries and mental health disorders, leading to increased health care provision. [4,5,6,7,8]

In 2018, the Defense Health Agency (DHA) added 4 weight loss agents (phentermine, benzphetamine, diethylpropion, and phendimetrazine) to the TRICARE Formulary, following authorization from the 2017 National Defense Authorization Act. [9 Additionally, coverage of brand name and specially ordered medication (non-formulary) with prior authorization was expanded to include approved long-term therapies: liraglutide, lorcaserin, naltrexone/bupropion, orlistat, and phentermine/topiramate. [9] In 2021, semaglutide, a GLP-1 receptor agonist originally developed for diabetes management, was included in the list of covered medications following U.S. Food and Drug Administration (FDA) approval. [10,11,12,13,14]. While the DHA approved the agents for weight management, consensus indicates these agents remain adjunctive to a comprehensive lifestyle intervention, and prescribers must consider their compatibility with an individual service member’s professional duties and personal lifestyle. [9,10,13,15,16]

Given the limited available information on prescription weight loss medication usage among military members, it is important to explore their prevalence to understand obesity management within the military. Describing their period prevalence serves as a precursor to further investigation of their real world effectiveness, side effects, and cost to the military health system. The objective of this descriptive epidemiologic study is to describe weight loss prescription medication prevalence among ACSM from January 2018 through June 2023.

## 2 METHODS

This report is based on summaries of medical administrative data routinely provided to the U.S. Armed Forces Health Surveillance Division (AFHSD) and integrated within the Defense Medical Surveillance System (DMSS) for health surveillance purposes. This retrospective cohort study included all active component U.S. military service members in the Army, Navy, Air Force, and Marine Corps between January 1, 2018 and June 30, 2023. Periodic Health Assessment (PHA), medical encounter, and demographic data were obtained from DMSS, the central repository of longitudinal medical surveillance data for directly and privately purchased medical care within the U.S. military. Records of prescribed and dispensed weight loss medications from the Pharmacy Data Transaction Service (PDTS) were also obtained from DMSS.

All dispensed formulary and non-formulary weight loss medications covered by DHA were identified in PDTS through a drug name search for medications listed in **Table 1**; over-the-counter formulations were not analyzed. Both generic and trade names were searched, apart from Qsymia and Contrave, which were identified only by trade name to avoid capturing prescriptions for neurologic treatments. Quarterly prevalence was calculated as the number of service members with a dispensed weight loss medications during the quarter of interest per 100,000 service members in service at any point during the quarter.

Covariates included sex, age, service, race and ethnicity, rank, occupation, history of type 2 diabetes diagnosis, and body mass index (BMI). A case of diabetes was defined by a record of 2 or more inpatient or outpatient medical encounters within 90 days of each other, with a diagnosis of type 2 diabetes mellitus in the first (primary) diagnostic position (International Classification of Diseases, 9th Revision [ICD-9]: beginning with ‘250’ and the fifth digit is ‘0’ or ‘2’; International Classification of Diseases, 10th Revision [ICD-10]: E11). Cases with a prior diagnosis for type 1 diabetes (ICD-9: beginning with ‘250’ and the fifth digit is ‘1’ or ‘3’; ICD-10: E10*) listed in the primary diagnostic position were excluded as a case of type 2 diabetes, as the type could not be determined.

To calculate BMI, records of height and weight were obtained from annual electronic PHA documentation. The highest weight measurement within the 2 years prior to the medication dispensation date was included to describe the maximum recorded BMI for service members dispensed a weight loss medication in the last quarter of the surveillance period. Weight records for women with a pregnancy or birth-related diagnosis (ICD-10 code beginning with ‘O’) in any diagnostic position in an inpatient or outpatient record within 9 months before or after the date of their weight measurement, were excluded from the analysis. BMI was calculated in kg/m^2^ [(Weight (lbs.) / (Height (in))^2^) * 703].

## 3 RESULTS

**Figure 1:**
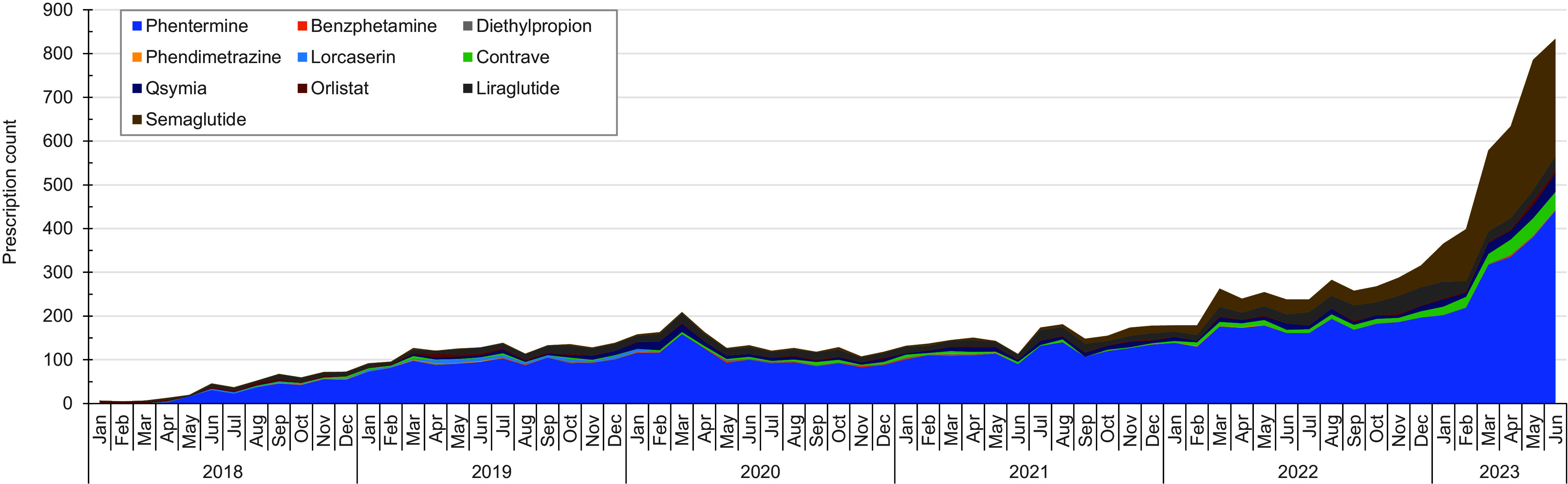
Weight Loss Prescription Counts in the Military Health System Among the Active Component, U.S. Armed Forces, 2018-2023

The number of monthly prescriptions of weight loss agents in the active component increased from 7 prescriptions in January 2018 to 816 prescriptions in June 2023 (**Figure 1**). Phentermine constituted the largest number of prescriptions throughout the surveillance period, while semaglutide comprised a significant proportion following its FDA approval in June 2021. The vast majority of prescriptions (n=12,037, 99.9%) during the study period were dispensed with a supply of 90 days or less.

**Figure 2:**
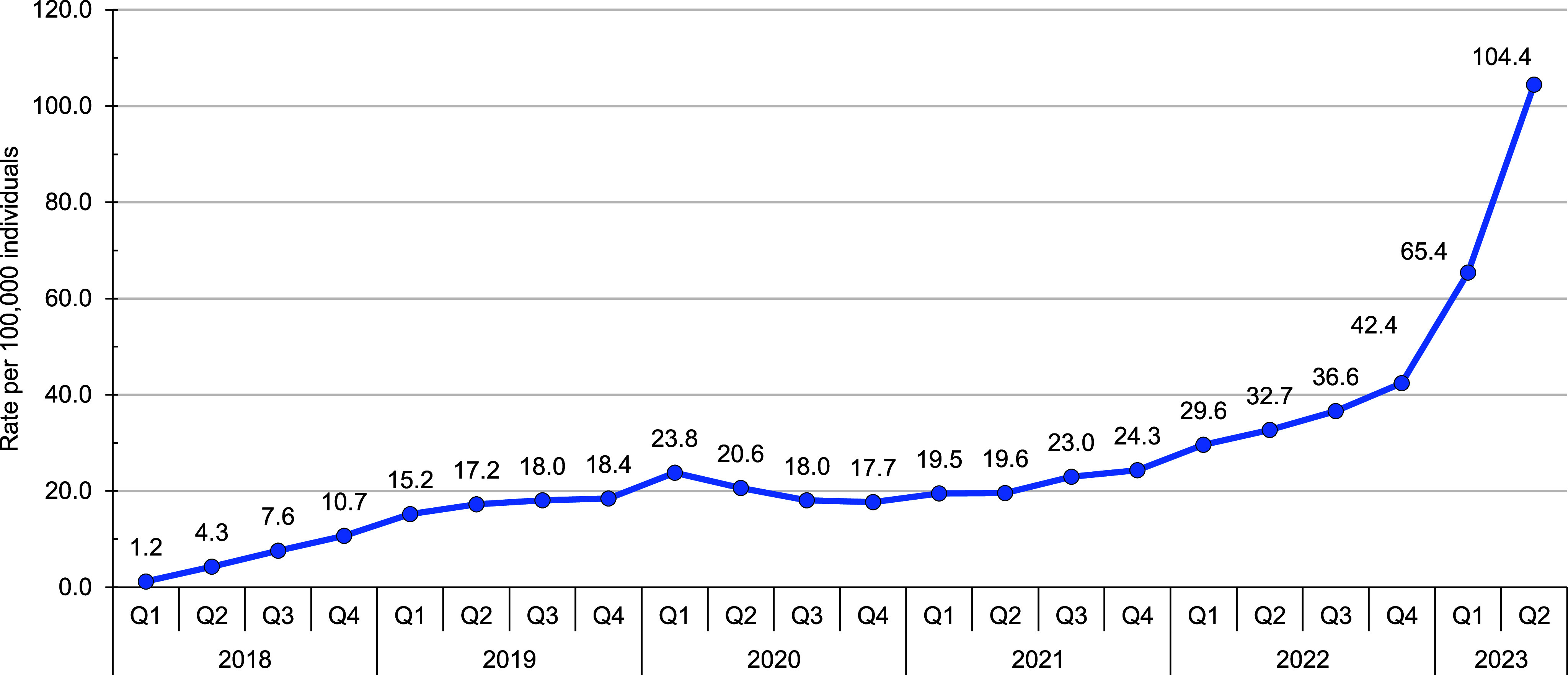
Weight Loss Prescription Prevalence Among the Active Component, U.S. Armed Forces, 2018-2023^a^

Initial analysis by agent revealed similar prevalence trends by demographic category (data not shown) and were collapsed to summarize findings as prevalence of all weight loss medications. The quarterly prevalence of ACSM who received any weight loss prescription (per 100,000 persons) increased from 1.2 in the first quarter (Q1) of 2018 to 104.4 in the second quarter (Q2) of 2023 (**Figure 2**). Female service members received prescriptions for weight loss at a higher rate than their male counterparts during the surveillance period, with a rate of 339.2 compared to 54.5 in Q2 of 2023 (**Table 2**). Prevalence rates increased with increasing age; those in the 50 years and older category had a rate of 470.6 in Q2 of 2023. Stratification by age and sex clearly delineated higher prevalence among women in all age groups than men of the same age, with an increasing prevalence with increased age.

Prescription prevalence was consistently higher among Navy ACSM than among other services, at 157.1 in Q2 of 2023, 72% higher than those in the Army (91.3), 54.5% higher than those in the Air Force (101.7), and 363% higher than the Marine Corps (43.2).

After excluding those whose race or ethnicity was ‘Unknown,’ non-Hispanic Black ACSM had the highest prescription prevalence, at 147.3 in Q2 of 2023. By comparison, non-Hispanic White ACSM had a prevalence of 95.9, Hispanic ACSM 93.0, and the “Other” race and ethnicity ACSM category 86.4 per 100,000 in Q2 of 2023. Stratified analysis by race and ethnicity and age showed highest prevalence among non-Hispanic Black personnel in all age categories, with the exception of Hispanic personnel in the 25-39 category.

Covariate analysis showed increasing prevalence of prescriptions. By rank, senior officers and senior enlisted ACSM had the highest prescription prevalence, reaching their highest rates in Q2 of 2023, 307 and 145.7 per 100,000 respectively. Comparing occupations, ACSM in a health care field had the greatest prescription prevalence over the study period, while those in pilot and air crew positions maintained lowest prevalence. Service members with a prior history of diabetes had significantly higher prescription prevalence, at 2,124.6 per 100,000 in Q2 of 2023.

Data (not shown) from Q2 of 2023 revealed that 62.1% of weight loss agents were prescribed to those with obesity (BMI ≥ 30 kg/m^2^); 16.9% of prescribed agents were for those with an overweight BMI (25–30 kg/m^2^) and 2.1% of agents were prescribed to those with a BMI less than 25 kg/m^2^. 18.9% of personnel on weight loss prescriptions did not have a calculable BMI from the corresponding PHA.

## 4 DISCUSSION

The U.S. Preventive Service Task Force reports that lifestyle change programs are evidence-based and should remain the primary focus for weight loss in adults while data on long-term weight maintenance after discontinuation of weight loss agents are lacking. [17] The current joint Department of Veterans Affairs and Department of Defense Clinical Practice Guidelines for weight loss medication administration require that patients start lifestyle modifications (i.e., physical training programs and reduced caloric intake) prior to initiating a weight loss medication. After 12 weeks of phentermine use, or in the presence of a contraindication to its use, a patient may be moved to a non-formulary weight loss agent. [18]

The results of this study demonstrate a substantial increase in the number of monthly prescriptions of weight loss agents among ACSM since initial approval in September 2017, with a 4-fold increase in prescribing rates starting in 2022. Phentermine consistently constituted the largest number of prescriptions throughout the study period, which is similar to previous studies of weight loss medication use in civilian populations. [19] The introduction of semaglutide following FDA approval in June 2021, however, resulted in increases in that agent’s prevalence, but its increase is not consistent with linear adoption patterns in the general U.S. population, and may be a result of the sequential process of obtaining semaglutide for weight loss in the military and inter-service variability in prescribing practices. [20] Alternatively, such results may demonstrate secondary effects on prescribing practices and treatment of obesity as a chronic disease as obesity prevalence increases within the Armed Forces and service members and providers become more aware of treatment options involving these medications.

The demographic profile of weight loss prescription ratios corresponds to previous observations of demographic factors associated with obesity prevalence in the U.S. military. [3] Non-Hispanic Black and older (40+) service members received weight loss prescriptions at higher rates than their counterparts, which is consistent with findings in the general U.S. population [19,21]. Navy ACSM consistently maintained higher prescription prevalence compared to all other service branches, which correlated with the Navy’s higher rates of obesity compared to all other services. [3]

There were some differences between the previous surveillance of obesity and prevalence of weight loss prescriptions identified in this study. Women had higher prevalence of weight loss medications despite a higher prevalence of obesity among men in the military. [3] Service members in health care fields had the highest prescription prevalence throughout the study period although obesity is more prevalent in repair/engineering occupations. [3] These findings may indicate the influence of professional factors and health care knowledge on medication use, general comfort with the concept of using a medication for weight management, improved access to medications through system or institutional knowledge, or increased awareness of overweight or obese status among health care workers. The increased prevalence of dispensed weight loss medications among service members in health care occupations requires further study.

This analysis relied on electronic health records, which may have inherent biases and limitations, including missing or incomplete data and data entry inaccuracies. Data from PHAs may not truly reflect accurate height and weight data; many health care providers conducted PHAs during the COVID-19 pandemic via telehealth, resulting in self-reported data that more commonly results in weight underestimation. Additionally, BMI is an imperfect measure for distinguishing lean versus fat body mass. While the TRICARE health benefit is intended to be the primary health care coverage for all ACSM, there is the infrequent possibility a service member acquires weight loss medications outside the MHS, which would not appear in this analysis. Over-the-counter formulations of the covered weight loss medications were not considered in this analysis, resulting in estimates that may be lower than true prevalence. Additionally, this study focused on ACSM, limiting the generalizability of the findings to other military populations or civilian contexts.

The U.S. Centers for Disease Control and Prevention estimated in 2022 that increased obesity prevalence in military members costs a potential $1.5 billion annually in obesity-related health care and 658,000 lost work days. [8] The trend in use of weight loss medication in the military should continue to be monitored, as these therapies represent a novel tool to manage obesity in this population. Future efforts should evaluate weight loss medications’ real world effectiveness, effects on health outcomes and comorbidities, safety for use in austere and deployed environments, impacts on readiness and retention, and cost-benefit and utility analyses.

## Figures and Tables

**Table 1:** Prescription Weight Loss Medications Analyzed

**Generic name**	**Trade name**	**Special prior authorization requirements**
Phentermine HCL	*Lomaira - 8mg (non-formulary)*	---
	Adipex-P - 37.5mg (formulary)	---
Benzphetamine HCL	Didrex (brand name discontinued)	---
	Regimex (brand name discontinued)	---
Diethylpropion HCL	Tenuate (brand name discontinued)	---
	Dospan	---
Phendimetrazine Tartrate	Phendiet	---
	Melfiat	---
	Anorex-SR	---
	Pleine	---
	Bontril	---
Lorcaserin	*Belviq*	---
	Belviq XR	---
Naltrexone / Bupropion SR	*Contrave*	Failure to lose weight on phentermine or has a contraindication its use.
Orlistat	*Xenical*	Failure to lose weight or contraindication to use phentermine or Contrave.
Phentermine / Topiramate ER	*Qsymia*	Failure to lose 5% of baseline weight with 12 weeks of phentermine use.
Liraglutide	*Saxenda*	Tried and failed or has a contraindication to ALL of the following: phentermine, Qsymia, and Contrave.
Semaglutide	*Wegovy*	Tried and failed or has a contraindication to ALL of the following: phentermine, Qsymia, and Contrave.
	Ozempic	

**Note: Italicized trade names are non-formulary within the Military Health System.**

**Table 2:** Second Quarter 2023 Prevalence of Dispensed Weight Loss Agents Among the Active Component, U.S. Armed Forces


No.	Rate^a^
Total	1,337	104.4
Sex		
Male	575	54.5
Female	762	339.2
Age group, years		
<20	0	0.0
20-24	89	22.3
25-29	190	62.6
30-34	251	117.9
35-39	352	217.1
40-44	269	319.1
45-49	113	358.6
50+	73	470.6
Service		
Army	419	91.3
Navy	515	157.1
Air Force	330	101.7
Marine Corps	73	43.2
Race and ethnicty		
Non-Hispanic White	655	95.9
Non-Hispanic Black	307	147.3
Hispanic	223	93.0
Other	113	86.4
Unknown	39	211.8
Rank		
Junior enlisted (E1-E4)	159	30.02
Senior enlisted (E5-E9)	756	145.7
Warrant officer (W01-WO5)	27	144.2
Junior officer	133	101.8
Senior officer	260	307.0
Unknown	2	247.2
Military occupation		
Combat-related^b^	55	31.2
Armor/motor transport	20	53.2
Pilot/air crew	10	21.8
Repair/engineering	228	62.3
Communications/intelligence	334	120.1
Health care	475	445.4
Other	215	79.7
History of type 2 diabetes		
No history	1,278	100.00
Previous diagnosis	59	2,124.6

^a^Rate per 100,000 individuals.

^b^Infantry/artillery/combat-engineering/armor.

## AUTHOR AFFILIATIONS

Uniformed Services University of the Health Sciences, Bethesda, MD: LT Lorei; Armed Forces Health Surveillance Division, Silver Spring, MD: Dr. Stahlman, Mr. Oh, CAPT Wells

## DISCLAIMER

The opinions and assertions expressed herein are those of the authors and do not reflect the official policy nor position of the Uniformed Services University of the Health Sciences, the Department of Defense, or the U.S. Government.

References to non-Federal entities or products do not constitute nor imply a Department of Defense or Uniformed Services University of the Health Sciences endorsement.

This work was prepared by a military or civilian employee of the U.S. Government as part of that individual’s official duties and therefore is in the public domain and does not possess copyright protection. Public domain information may be freely distributed and copied; however, as a courtesy it is requested that the Uniformed Services University and the author be given an appropriate acknowledgment.
